# Support for targeted sampling of red fox (*Vulpes vulpes*) feces in Sweden: a method to improve the probability of finding *Echinococcus multilocularis*

**DOI:** 10.1186/s13071-016-1897-3

**Published:** 2016-11-29

**Authors:** Andrea L. Miller, Gert E. Olsson, Sofia Sollenberg, Moa Skarin, Helene Wahlström, Johan Höglund

**Affiliations:** 1 Department of Biomedical Sciences and Veterinary Public Health, Section for Parasitology Swedish University of Agricultural Sciences, Box 7036, Uppsala, 750 07 Sweden; 2 Department of Wildlife, Fish, and Environmental Studies, Swedish University of Agricultural Sciences, Umeå, 901 83 Sweden; 3 Department of Epidemiology and Disease Control, Zoonosiscenter, National Veterinary Institute (SVA), Uppsala, 751 89 Sweden

**Keywords:** Foxes, Feces, Public health, Sweden, Epidemiology, *Echinococcus multilocularis*, Alveolar echinococcosis, Risk-based sampling, Targeted sampling

## Abstract

**Background:**

Localized concentrations of *Echinococcus multilocularis* eggs from feces of infected red fox (*Vulpes vulpes*) can create areas of higher transmission risk for rodent hosts and possibly also for humans; therefore, identification of these areas is important. However, in a low prevalence environment, such as Sweden, these areas could be easily overlooked. As part of a project investigating the role of different rodents in the epidemiology of *E. multilocularis* in Sweden, fox feces were collected seasonally from rodent trapping sites in two regions with known parasite status and in two regions with unknown parasite status, 2013–2015. The aim was to evaluate background contamination in rodent trapping sites from parasite eggs in these regions. To maximize the likelihood of finding fox feces positive for the parasite, fecal collection was focused in habitats with the assumed presence of suitable rodent intermediate hosts (i.e. targeted sampling). Parasite eggs were isolated from feces through sieving-flotation, and parasite species were then confirmed using PCR and sequencing.

**Results:**

Most samples were collected in the late winter/early spring and in open fields where both *Arvicola amphibius* and *Microtus agrestis* were captured. Fox feces positive for *E. multilocularis* (41/714) were found within 1–3 field collection sites within each of the four regions. The overall proportion of positive samples was low (≤5.4%) in three regions, but was significantly higher in one region (22.5%, *P* < 0.001). There was not a significant difference between seasons or years. Compared to previous national screenings, our sampling strategy identified multiple *E. multilocularis* positive feces in all four regions, including the two regions with previously unknown parasite status.

**Conclusions:**

These results further suggest that the distribution of *E. multilocularis* is highly aggregated in the environment and provide support for further development of a targeted sampling strategy. Our results show that it was possible to identify new areas of high contamination in low endemic environments. After further elaboration, such a strategy may be particularly useful for countries designing surveillance to document freedom from disease.

**Electronic supplementary material:**

The online version of this article (doi:10.1186/s13071-016-1897-3) contains supplementary material, which is available to authorized users.

## Background

**Fig. 1:**
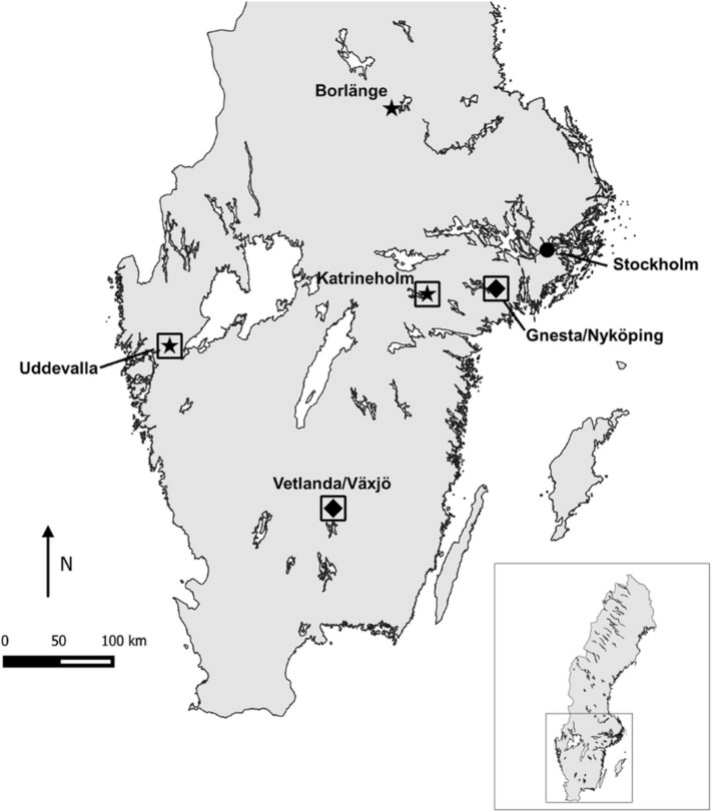
Map showing the southern half of Sweden and study regions (boxes). Black stars indicate areas where intestinal samples from shot foxes were identified as positive for *Echinococcus multilocularis* through national monitoring (2011) before this study began (2013) [4]. Black diamonds indicate additional areas identified positive for *E. multilocularis* by the conclusion of this study (2015). Map created in QGIS v2.12.3. (Basemap: Sweden 1000plus 6.0, SWEREF 99 TM, 2008, © Lantmäteriet). Modified from Fig. 1 in Miller et al. [5]

*Echinococcus multilocularis*, a zoonotic parasite of wildlife, is considered an emerging disease in Europe. Its spread and increasing incidence have been cited for many reasons including increasing trade and travel of untreated dogs, increasing definitive and intermediate host populations, and increasing awareness by the public and public health authorities [1]. Although the occurrence in humans is rare, the disease is usually fatal without treatment and treatment, itself, is long-term, potentially invasive, and costly [2]. In response to the parasite’s increasing geographic range, national authorities in Sweden began monitoring for *E. multilocularis* in red fox (*Vulpes vulpes*) in 2000 [3]. After nearly ten years of monitoring, *E. multilocularis* was first identified in a red fox shot on the west coast of Sweden in 2010 [3]. This finding prompted a survey of intestinal samples from red foxes collected nation-wide. From these results, three positive foxes (out of 2985 examined) in three different regions (Borlänge, Katrineholm, Uddevalla) (Fig. 1) were identified, and the prevalence of *E. multilocularis* was estimated to be ~0.1% on a national level [4]. However, questions still remained about the true parasite distribution, the role of the intermediate hosts, and the transmission dynamics on a local level.

Transmission of *E. multilocularis* depends on a complex interaction between the parasite’s canid definitive hosts, its rodent intermediate hosts, and environmental factors. In Sweden, the red fox is considered the major definitive host [3], and early results indicate that rodent intermediate hosts include both the field vole (*Microtus agrestis*) and the water vole (*Arvicola amphibius*) [5]. Foci of high numbers of infected foxes are reported in many countries, including Germany [6] and France [7]. Infected foxes shed eggs into the environment through feces. While foxes may defecate anywhere, their defecation behaviors tend to reflect local access to food resources and territorial markings [8, 9]. For instance, studies in France have demonstrated high fox fecal density in areas of high rodent density compared to areas with lower rodent density [10, 11]. Within these foci of high *E. multilocularis* prevalence in foxes, aggregations of infected feces create areas with high levels of parasite eggs in the environment. These areas may only be a matter of hundreds of square meters and have been termed “micro-foci” [12].

Transmission between the definitive and intermediate hosts are facilitated within these micro-foci. The risk of transmission is subject to a number of influences, such as temperature and humidity (egg survival), host susceptibility, host density, and host behavior [13]. Optimal conditions for parasite transmission in western Europe have been described to include high densities of infected foxes feeding on high densities of susceptible and easily accessible intermediate hosts in grassland habitats [13]. These same foxes are ideally shedding high numbers of eggs through their feces deposited within susceptible rodent intermediate host habitats [13]. Optimal egg survival would occur in feces shed through the winter and/or in moist micro-habitats [13]. As humans become infected through accidental ingestion of parasite eggs, these micro-foci represent an increased transmission risk for not only rodent intermediate hosts, but likely also for humans [12]. Therefore, to better assess the risk for human exposure, a better understanding of the distribution of parasite eggs and of the factors contributing to this distribution in the environment is needed.

Risk-based sampling is considered an efficient method of disease detection, particularly for diseases with low prevalence [14]. This type of sampling is focused on populations and/or environments where the probability of disease is higher [14]. To determine high-risk population/environments, clearly defined risk factors for disease presence are needed [14]. For example, a study proposing a risk-based model for sampling production pigs in Denmark for *Trichinella* spp. defined pigs housed outdoors as animals at high risk for exposure to the parasite [15]. Such criteria are not easily defined for *E. multilocularis*, which has a complex life-cycle in wildlife influenced by many intrinsic and extrinsic factors. Despite this, the use of risk-based sampling for *E. multilocularis* to document freedom from disease has been suggested in a recent scientific opinion by EFSA [16]. In this study, the term targeted sampling was used instead, as risk factors used could not be clearly defined [14].

This project began in 2013 and was designed to describe the role of the rodent in the life-cycle of *E. multilocularis* in Sweden. As the rodent intermediate host(s) was yet unknown, the primary purpose was to identify the rodent host and to describe characteristics of the parasite infection within these hosts in Sweden [5]. Because parasite prevalence in foxes was estimated to be extremely low (0.1%), sampling was designed to maximize the likelihood of finding the parasite and considered the optimal conditions for transmission outlined above [13]. In particular, we targeted fields with signs of the most likely rodent intermediate hosts, field voles and water voles. The aim of this paper is to describe the local level of environmental contamination of *E. multilocularis* eggs using fox feces collected in limited areas surrounding rodent trapping sites from four different regions in southern Sweden. Two of these regions had a known parasites status and two had unknown parasite status at the onset of the study. These findings, in light of the study design, are discussed as a basis for future risk-based sampling of *E. multilocularis*.

## Methods

### Study regions

Fox feces were collected during 2013–2015 as part of a research project investigating *E. multilocularis* in rodents in Sweden [5]. Collections occurred within four study regions within the municipalities of Katrineholm, Uddevalla, Gnesta/Nyköping, and Vetlanda/Växjö (Fig. 1). The regions of Katrineholm and Uddevalla (~10 × 10 km) were selected as they were regions where *E. multilocularis* had been previously identified in the initial national screening of hunter shot foxes in 2011 [4]. The regions of Gnesta/Nyköping and Vetlanda/Växjö (~20 × 20 km) were selected for practical reasons as they were part of the Environmental Monitoring and Assessment at the Swedish University of Agricultural Sciences (FoMA, http://www.slu.se/en/environment) where seasonal rodent trapping had been occurring for other purposes since 2012. As *E. multilocularis* had not been identified in the FoMA regions in the 2011 national surveillance, the *E. multilocularis* status in foxes in these regions was unknown at the beginning of the study. All study regions were located in the south of Sweden because the fox density was estimated to be higher in the south than in the north and because no positives had been found north of Borlänge (60.48°N, 15.43°E) [4]. For a more detailed description of field design and rodent trapping methods see Miller et al. [5].

### Fecal collection

Fox fecal collection was focused on or near rodent trapping sites. The targeted rodent species were water voles, field voles and bank voles (*Myodes glareolus*). These are species with a wide geographical range in Sweden and which are closely related to species reported to have a high prevalence of *E. multilocularis* in central Europe [5, 17, 18]. Rodent trapping sites were selected based on the following criteria: expert knowledge of preferred habitat for the targeted rodent species, presence of rodent activity (i.e. signs of tunnels and tumuli of field or water voles), nearness to an ecotone (i.e. an area with potentially higher species diversity [19]), prior knowledge of *E. multilocularis* findings (within positive regions), and, to a lesser extent, logistics (i.e. accessibility). Particular focus on fecal collection was spent within field habitat where field voles and water voles were trapped, as these species were *a priori* considered the most likely of the three targeted species to be potential intermediate hosts [5]. Although these field habitats varied, field and water voles were most often trapped in unplowed grassy areas near an irrigation ditch, stream, or other source of water. For the purposes of this paper, a fecal “collection site” is defined as any area where at least one fox feces was found and which was on or near (~500–600 m) a rodent trapping site.

**Fig. 2:**
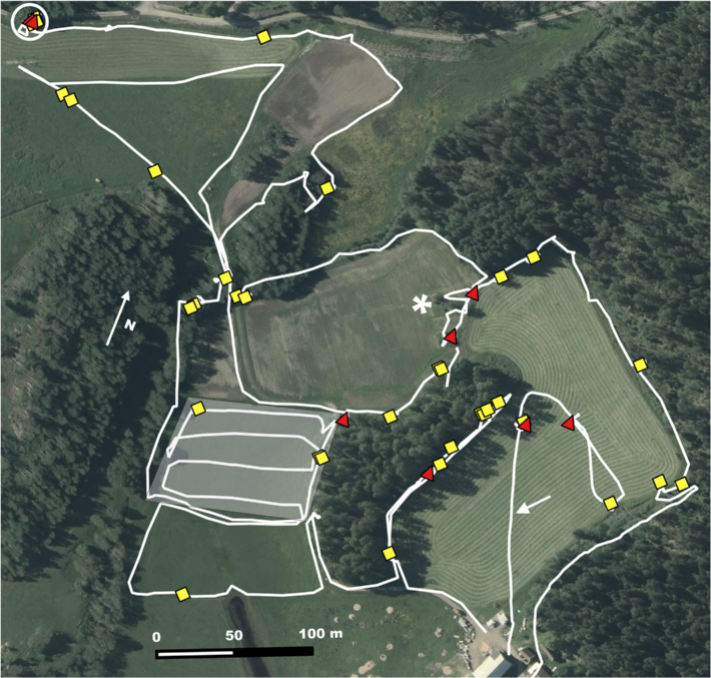
Map demonstrating the search pattern used for sampling of fox feces in a collection site March 2015. The white line depicts the GPS tracklog (walking path) of the researcher. Yellow diamonds are *Echinococcus multilocularis* negative feces and red triangles are *E. multilocularis* positive feces. Some landscape features, which were used to direct the search pattern, are labeled on the map. The white arrow (low center at right) indicates a track along a mowed grass path not shown on map. The white asterisk (center) indicates an area of stones. The grey shaded area (left-center) is an area of very dense water vole activity and indicates one area where these rodents were trapped. The white circle (at top left) indicates a well top. A North arrow is present far left, outside the sampling area. Map created in QGIS v2.12.3 with a background satellite image (WMS ortofoto årsvis 2014, SWEREF99, © Lantmäteriet)

To find feces, we followed anthropogenic ecotones such as field/forest edges, fence rows, ditches, but also natural game trails and/or examined fox marking sites such as water vole mounds, water well covers, footbridges, and elevations in the landscape (Fig. 2) [20, 21]*.* Feces were identified as fox feces based on appearance (e.g. shape and size), and location in the environment (e.g. top of rock) [20]. Feces were collected wearing disposable plastic gloves and were immediately put into plastic fecal tubes (Sarstedt, Nübrecht, Germany). Georeferences were obtained by handheld GPS units (Garmin, Kansas, USA) for each fecal sample collected.

Fecal collections corresponded to the rodent trapping periods, which occurred seasonally in spring (April-June) and autumn (September-October) beginning spring 2013 and ending spring 2015. During these times, feces were collected opportunistically if observed while trapping. In addition, feces were also collected before spring rodent trapping during winter 2014 (February-March) and winter 2015 (March-April). During the winter collections, 4–7 field collection sites, known for previous successful fecal collections and/or rodent captures, were selected in each region to allow for a more systematic and focused collection effort (Fig. 2). Winter collections always occurred after snowmelt but prior to onset of the grass vegetation period, after which accumulated overwinter feces could have been overgrown and more difficult to find.

### Collection of parasite eggs

For biosecurity reasons, all fecal samples were frozen at -80 °C for at least one week before analysis. After thawing, eggs were isolated from two grams of mixed feces using the sieving-flotation procedure as described in Mathis et al. [22]. The only modification was a preliminary step whereby feces were incubated in PBS (1:4) in the refrigerator (4 °C) overnight. Samples were then frozen at -20 °C until molecular analysis.

### Molecular analyses

Only samples PCR-positive and confirmed *E. multilocularis* through sequencing were considered as *E. multilocularis*-positive. The sample pellet was first broken through the alkaline lysis and neutralization step outlined in Mathis et al. [22]. DNA was then extracted following the procedure outlined in Štefanić et al. [23] using the QIAamp® DNA mini kit (Qiagen, Hilden, Germany). Similar to our previous study [5], parasite species were identified using a multiplex PCR with primers specific for *E. multilocularis*, *E. granulosus* and *Taenia* spp. targeting the NADH dehydrogenase subunit 1 gene (*nad1*) of the mitochondrial DNA [24]. PCR products from observed bands were purified using the Illustra ExoProStar 1-step kit (VWR International, PA, USA), or, in cases where two bands were present, the QIAquick® Gel Extraction Kit (Qiagen, Hilden, Germany) and sent for sequencing (Macrogen, Amsterdam, The Netherlands). Sequence quality was analyzed using CLC Main Workbench v5.6.1 (CLC Bio) and submitted for a nucleotide identity match using the Basic Local Alignment Search Tool (BLAST) through the NCBI database [25]. Sequences were then imported into Mesquite v3.04 [26] and automatically aligned in MAFFT v7.0 [27] using the default settings together with representative *nad1* sequences for *E. multilocularis*, *E. granulosus*, *E. canadensis*, *E. equinus* available in GenBank [28]. Sequences were trimmed to match the primers and compared after being finally aligned manually.

### Statistical analysis

All statistical analyses were performed in R v3.2.2 [29]. Because the “winter” months overlapped with the “spring” months, feces collected from both these periods were combined into one “winter/spring” period for seasonal analysis. As the sampling seasons varied each year, comparisons between years was limited to data collected in the same seasons (i.e. spring/fall 2013 and spring/fall 2014; 2014 winter and 2015 winter). The proportions and 95% CI of feces positive for *E. multilocularis* were calculated for site, region, season, and year using the *BINOM* package [30]. Graphs were produced using GraphPad Prism 5 (GraphPad Software, La Jolla, California, USA).

To compare the differences between study regions, seasons, and years, a logistic mixed model with region, season, and year as fixed factors and collection site within region as a random variable was considered. However, the dataset (Additional file 1: Table S1) was unbalanced and contained a relatively small number of positive samples. This created poorly fitted models and, consequently, large uncertainty in the resulting *P*-value estimates. Therefore, univariate analyses were performed to compare differences between study regions, seasons, and years using the Fisher’s exact test of independence [31]. If the initial analysis was significant (*P* ≤ 0.05), pair-wise Fisher’s exact tests were used to distinguish between the different combinations of factors (e.g. regions: Katrineholm, Uddevalla, Gnesta/Nyköping, Vetanda/Växjö). To account for multiple tests performed, a Bonferroni correction was used [31].

## Results

### Fecal collection results

A total of 714 fecal samples (Uddevalla, *n* = 336; Katrineholm, *n* = 189; Vetlanda/Växjö, *n* = 109; Gnesta/Nyköping, *n* = 80) were collected and analyzed over seven collection periods (2013–2015) for the presence of *E. multilocularis* DNA (Additional file 1: Table S1). These 714 feces were collected from 57 fecal collection sites (Additional file 2: Figure S1). The number of feces collected varied from one to 92 per collection site (Additional file 2: Figure S1). Nearly all feces (685/714, 96%) were collected from open/field habitat or from forest/field edges. The remaining 29 (4%) were collected from forest habitat.

More feces (628/714, 88%) were collected in the winter/spring season than in the fall (86/714, 12%). Of the 714 samples, 229 (32%) were collected during rodent trapping and 485 (68%) were collected before rodent trapping (winter collections). Due to logistical constraint, almost all feces in the FoMA sites (Gnesta/Nyköping 63/80, 79%; Vetlanda/Växjö 100/109, 92%) were collected before rodent trapping.

### *Echinococcus multilocularis* results

Forty-six of 714 feces (6.4%) were PCR-positive for *E. multilocularis*. However, a 344 bp fragment of *nad1* (including substitutions but excluding the primer sites) could be successfully amplified from only 41 samples. Therefore, only 41/714 (5.7%, 95% CI: 4.2–7.7%) samples were considered *E. multilocularis* positive. Although nine sequences were of poor quality and/or incomplete, all 41 sequences were matched highly to *E. multilocularis*. When aligned, the 32 full length, high quality sequences were identical to each other and matched previously identified *E. multilocularis* haplotypes (e.g. KF962559, AB668376, AY389984). They did not match *E. canadensis*, *E. granulosus* or *E. equinus* sequences.

**Fig. 3:**
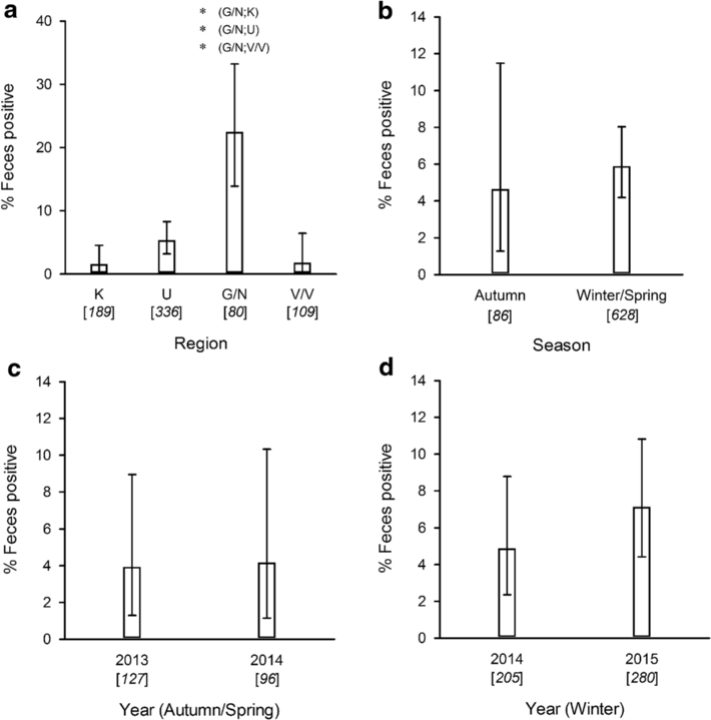
Proportion (in percentage) of feces positive for *Echinococcus multilocularis* by study region (**a**), season (**b**), and year (**c**, **d**). Comparisons between years are limited to those seasons which are repeated (**c**: autumn/spring; **d**: winter). The bars are binomial exact 95% CI. Sample size is indicated in parentheses under the x-axis. Study regions are K (Katrineholm), U (Uddevalla), G/N (Gnesta/Nyköping), and V/V (Vetlanda/Växjö). Significant differences (*P* < 0.001) are indicated by (*)

*Echinococcus multilocularis* was identified in all 4 study regions (Uddevalla: 18/336, 5.4%, 95% CI: 3.2–8.3%; Katrineholm: 3/189, 1.6%, 95% CI: 0.3–4.6%; Vetlanda/Växjö: 2/109, 1.8%, 95% CI: 0.2–6.5%; Gnesta/Nyköping: 18/80, 22.5%, 95% CI: 13.9–33.2%) (Fig. 3). Positive fecal samples were found all years in Uddevalla and in 2014 and 2015 in Gnesta/Nyköping, whereas positive feces were only found during 2013 in Katrineholm and only once in spring 2014 in Vetlanda/Växjö (Additional file 1: Table S1). *Echinococcus multilocularis* positive samples were only found in 1–3 of the 7–21 collection sites sampled within each region (Table 1; Additional file 2: Figure S1). The highest proportion of positive feces (13/25, 52%, 95% CI: 31.3–72.2%) was found in one collection site within Gnesta/Nyköping (Table 1).

**Table 1:** Description of collection sites containing feces positive for *Echinococcus multilocularis*, Sweden, 2013–2015

Region (*n*)	Collection site	Habitat^a^	Total feces	Pos. feces^b^	95% CI (%)	Rodents analyzed^c^	Pos. rodents^b^	95% CI (%)
K (18)								
	Site 1	Field	62	3	4.8 (1.0–13.5)	61	0	0 (0–5.9)
U (21)								
	Site 1	Field	92	15	16.3 (9.4–25.5)	43	0	0 (0–8.2)
	Site 2	Field	19	1	5.3 (0.1–26.0)	4	0	0 (0–60.2)
	Site 3	Field	63	2	3.2 (0.4–11.0)	52	0	0 (0–6.8)
G/N (7)								
	Site 1	Field	20	5	25.0 (8.7–49.1)	49	0	0 (0–7.3)
	Site 2	Field	25	13	52.0 (31.3–72.2)	79	6^d^	7.6 (2.8–15.8)
V/V (11)								
	Site 1	Field	37	1	2.7 (0.1–14.2)	2	0	0 (0–84.2)
	Site 2	Field	20	1	5.0 (0.1–24.9)	0^e^	0	0 (0–100)

*Abbreviations*: *n* total collection sites, *Pos.* number of positives, *95% CI* percent positive and 95% binomial exact confidence interval, *K* Katrineholm, *G/N* Gnesta/Nyköping, *U* Uddevalla, *V/V* Vetlanda/Växjö

^a^The habitat (forest or field) that covered the majority of the collection site

^b^Number of feces or rodents positive for *Echinococcus multilocularis*

^c^Number of rodents caught within the collection site and analyzed for *Echinococcus multilocularis*. The majority of rodents analyzed from these sites were either water voles (*Arvicola amphibius*) or field voles (*Microtus agrestis*) but could include mice (*Apodemus* spp.) and bank voles (*Myodes glareolus*). Based on a previous study [5]

^d^Five water voles (*A. amphibius*), one field vole (*M. agrestis*)

^e^Although traps were set out, no rodents were caught

The proportion of positive samples was significantly different (*P* < 0.001) between regions, but not between seasons (*P* = 0.807) or years (autumn/spring 2013/2014: *P* = 1.000; winter 2014/2015: *P* = 0.345) (Fig. 3). Results of the pairwise comparisons among the regions are presented in Fig. 3. Using the Bonferroni correction for multiple tests, only the proportion of *E. multilocularis* positive samples in Gnesta/Nyköping was significantly different from the other sites.

## Discussion

### Spatial and temporal distribution

Although positives were found in all study regions, the proportion of *E. multilocularis* positive feces in Gnesta/Nyköping was significantly different. This difference was evident despite the small sample size (*n* = 80) within this study region. This, in addition to the fact that positive feces were limited to a few areas per region, provide evidence of a highly aggregated distribution of *E. multilocularis* in Sweden. These results support similar findings from our previous rodent study [5].

The positive collection sites within Gnesta/Nyköping were similar in that each contained a high number of feces (> 15 samples collected) associated with field habitat where high numbers of both field and water voles were trapped. The individual collection site with the highest proportion of positive feces (52.0%, 2013–2015) in Gnesta/Nyköping was also the collection site with the highest proportion of positive rodents (6/79, 7.6%, 2013–2015) found earlier in this research (Table 1) [5]. This provides evidence that a high density of positive feces and presence of suitable rodent intermediate hosts, particularly in field habitat, are important transmission factors. However, the collection site with the highest proportion of positive feces in Uddevalla (16.3%, 2013–2015) contained no positive rodents (0/43, 0%, 2013–2014) (Table 1) [5]. Because the study design and data collection herein did not include specific habitat variables (e.g. soil type, plant species) or allow for standardized estimates of rodent or fecal density, it was not possible to statistically model the differences between these collection sites accurately. Therefore, these observations should highlight the need for further investigation into microhabitat and other factors that may attract foxes and/or facilitate parasite transmission to suitable rodents.

The percentages of positive feces presented in this paper should not be interpreted as *E. multilocularis* prevalence in foxes. These percentages are rather an estimate, or index, of local environmental contamination [32]. A focused collection of fox feces in a small area is likely to collect samples from the same individual. Still, as positive samples are reported from different collection sites, regions, and years, it seems highly unlikely that all 41 samples originated from the same fox. In addition, morphological species identification of feces is not precise. It cannot be excluded that some feces could have been misidentified for such species as domestic dogs, cats, or mustelids (e.g. pine marten *Martes martes*, least weasel *Mustela nivalis*, stoat *Mustela erminea*) [20]. Of these, only foxes, and, to a much lesser extent, dogs are likely to host *E. multilocularis* [33]. To the authors’ knowledge, there is only one report of mustelids (i.e. *Martes* spp. in Russia) hosting *E. multilocularis* [34]. Cats may also be infected; however, cats are considered poor hosts due to low infection intensity and few infective eggs produced [33]. Recent studies have used molecular methods to confirm species identification of feces [35, 36], but these methods were not used here as background environmental contamination from feces occurs regardless of the definitive host. Although misidentified feces may have led to an underestimation of the positive proportions, this underestimation would not change the conclusions drawn from the results. In fact, if higher proportions could be expected, it would only strengthen the differences seen between collection sites and between sampling designs (as discussed in the next section).

Irrespective of species and individual identity, the percentage of positive feces reported here reflect areas of concentrated egg contamination in the Swedish environment. Such micro-foci are considered as high risk areas for *E. multilocularis* transmission to suitable rodents and possibly also for humans. Increased incidence of human alveolar echinococcosis has been documented in areas with foci of highly infected definitive and intermediate host species [12], and these human cases can also be clustered into foci of infection [37]. Although there have been no autochthonous human cases in Sweden [38] and the estimated prevalence *E. multilocularis* in of foxes in Sweden is very low (0.1%) [4, 38, 39], the presence of such micro-foci suggest a need for continued research and monitoring for this parasite in Sweden.

Surprisingly, there was no statistically significant temporal variation in the *E. multilocularis* proportions between years or between seasons. Studies in Switzerland have identified higher numbers of positive foxes and positive fox feces in the late autumn/winter as compared to spring/summer [40, 41]. Furthermore, a study in Japan has reported yearly variation in prevalence of *E. multilocularis* in red fox to be associated with changes in the abundance and infection level of the rodent intermediate host [42]. In this study, it cannot be excluded that the low sample size, low number of positives and, thus, large uncertainty in the proportions reported have failed to identify any temporal trends present. However, it seems that no major variations occurred.

### Sampling considerations

For comparison, the major epidemiological investigations regarding *E. multilocularis* in Sweden, including this project (EMIRO), are summarized in Table 2. At the EMIRO project start in 2013, the national prevalence of *E. multilocularis* in foxes was estimated to be very low (0.1%) [4]. This estimation was further supported by a regional study based on fox feces surrounding a known infected area near Katrineholm (2011) which found an only slightly higher prevalence (0.8%) [38]. Therefore, when we began this project focused on rodent collection sites, we expected to find very few positive fox feces, particularly in the regions with an unknown *E. multilocularis* status. However, using the sampling strategy described herein, multiple positive feces were identified in all four study regions, two with a known parasite status (Katrineholm, Uddevalla) and two with an unknown parasite status (Gnesta/Nyköping, Vetlanda Växjö) in 2013. As such, the results of our sampling strategy reconfirmed the parasite presence in two regions, and identified *E. multilocularis* in two regions where parasite presence was unknown at study start.

**Table 2:** Summary of major investigations undertaken in Sweden to examine for *Echinococcus multilocularis* in red foxes (*Vulpes vulpes*) and in rodents

Investigation	Duration	Species/sample	*n*	Pos. (%)	Year	Place of positive finding	Reference
SVA							
Yearly monitoring	2000–2010	Fox intestines	3266	1 (<0.01)	2010	U	Osterman Lind et al. [3]
First nation-wide screening after positive finding	2011	Fox intestines	2985	3 (0.1)	2011	B, K, U	Wahlström et al. [4]
Regional survey^a^	2011	Rodent livers	236	0 (0)	2011		Wahlström et al. [4]
Regional survey^b^	2011	Fox feces^c^	790	6 (0.8)	2011	K	Wahlström et al. [38]
Second nation-wide screening	2012–2014	Fox feces^c^	2779	3 (0.1)	2012–2014	G/N, K, U	National Veterinary Institute (www.sva.se) [39]
SLU							
EMIRO project^d^	2013–2015	Rodent livers	1566	9 (0.6)	2013–2015	G/N, K	Miller et al. [5]
	2013–2015	Fox feces^c^	714	41 (5.7)	2013–2015	G/N, K, U, V/V	This paper

*Abbreviations*: *n* total samples, *Pos. (%)* number and percent positive, *SVA* National Veterinary Institute, *SLU* Swedish University of Agricultural Sciences, *EMIRO Echinococcus Multilocularis* in ROdents-this research project, *B* Borlänge, *K* Katrineholm, *G/N* Gnesta/Nyköping, *U* Uddevalla, *V/V* Vetlanda/Växjö

^a^Samples collected near Uddevalla

^b^Samples collected from a localized region (50 km diameter) near Katrineholm

^c^Feces collected from environment

^d^Samples collected from four regions (10 × 10 km or 20 × 20 km) in Sweden

During the completion of this project, a second nation-wide screening based on fox feces (2012–2014) was performed [39]. Compared to our findings (41/714, 5.7%, 95% CI: 4.2–7.7%), this second screening identified a significantly lower proportion of positives (3/2779, 0.1%, 95% CI: 0–0.3%; *P* < 0.001, Fisher’s test). The difference is also statistically significant (*P* < 0.001, Fisher’s test) when compared only to the two regions with an unknown status at study start (20/189, 10.6%, 95% CI: 6.6–15.9%). The national screening employed a newly designed magnetic-capture PCR [43] diagnostic technique with a reported sensitivity of 88% [43, 44], while the combined egg isolation and PCR technique used in this study has a lower reported sensitivity of 50% [45]. Thus, the difference cannot be explained by the diagnostic methods used. Therefore, it is suggested that the dissimilarity between these findings may be a result of the difference in collection methods.

The national screening for *E. multilocularis* in Sweden aimed to estimate the prevalence of *E. multilocularis* by using a systematic sampling method to collect representative samples from the whole country [38, 39]. This type of sampling makes no assumptions about the distribution of infected foxes (feces) in the country. However, results from the present study and others clearly show that *E. multilocularis* has a heterogeneous distribution in the environment and may be present in micro-foci [5–7, 12]. In a low endemic environment, large-scale and systematic sampling will likely miss micro-foci, as the results herein have demonstrated [32]. In addition, the sample size needed to detect a disease with a prevalence close to zero (i.e. 0.1%) with a confidence level of 95% is large (~3000) [32] and obtaining such sample numbers can be associated with a high cost [46].

In contrast to systematic sampling, risk-based sampling assumes a heterogeneous distribution of a disease and aims to maximize the likelihood of detecting disease by using prior knowledge of disease risk factors to focus the sampling efforts [14]. In low endemic countries, cost-efficient risk-based methods could be used to detect new areas of infection thereby improving the knowledge of parasite distribution. For instance, risk-based sampling could be used in the northern part of Sweden where *E. multilocularis* has never been detected before. Particularly for countries striving to document freedom from *E. multilocularis* (e.g. mainland Norway, Finland, Ireland and the UK), risk-based sampling could be expected to provide a more efficient method for detecting the parasite and allow for optimization of limited surveillance resources [16].

Fecal collections in this study were performed based on prior knowledge of risk factors for the presence of *E. multilocularis* known from the literature (i.e. [13]) and were specifically focused in habitats where rodent intermediate hosts deemed at high risk of hosting the parasite were abundant. Although this may be considered as risk-based sampling, we define our methods as targeted sampling. Targeted sampling has been used in a wider context than risk-based sampling [14]. As the risk criteria used for the sampling in this study were very broadly defined and could not be empirically tested (within the scope of this study), we instead chose to use this wording. For instance, the criteria “field” habitat is a very general definition for any number of habitats which may attract water or field voles and, consequently, a fox predator. Still, it is evident that more positive fox feces were found in this study than in the national screening - the only study to which the observations presented herein can be compared [39]. The success and applicability to larger areas of risk-based sampling requires clearly defined risk factors [14]. Therefore, the results of this study are an important first step in developing future risk-based sampling to identify *E. multilocularis* in a low endemic area and can serve as a basis for further research.

## Conclusion

The targeted sampling used in this study appears to be a more effective method to detect *E. multilocularis* in a low endemic environment. Using this sampling strategy, multiple positive feces and new areas of infection were detected.

## Additional files

Additional file 1: Table S1. Summary of fox feces collected by region, season, year and method. (XLSX 11 kb)
Additional file 2: Figure S1. Total number of fox fecal collection sites and number of feces collected within each site for each study region 2013–2015. (DOCX 139 kb)

## Abbreviations

95% CI: 95% confidence interval
BLAST: Basic Local Alignment Search Tool
EFSA: European Food Safety Authority
EMIRO: Echinococcus Multilocularis in ROdents research project
FoMA: Environmental Monitoring and Assessment at the Swedish University of Agricultural Sciences (Sweden)
G/N: Gnesta/Nyköping
K: Katrineholm
U: Uddevalla
V/V: Vetlanda/Växjö
