# Supplementary material for: Support for targeted sampling of red fox (Vulpes vulpes) feces in Sweden: a method to improve the probability of finding Echinococcus multilocularis
Source: Parasit Vectors. 2016 Nov 29;9:613. doi: 10.1186/s13071-016-1897-3 (PMC5129611; doi:10.1186/s13071-016-1897-3)
Supplement: Additional file 2: Figure S1. — Total number of fox fecal collection sites and number of feces collected within each site for each study region 2013–2015. (DOCX 139 kb) [file 13071_2016_1897_MOESM2_ESM.docx]

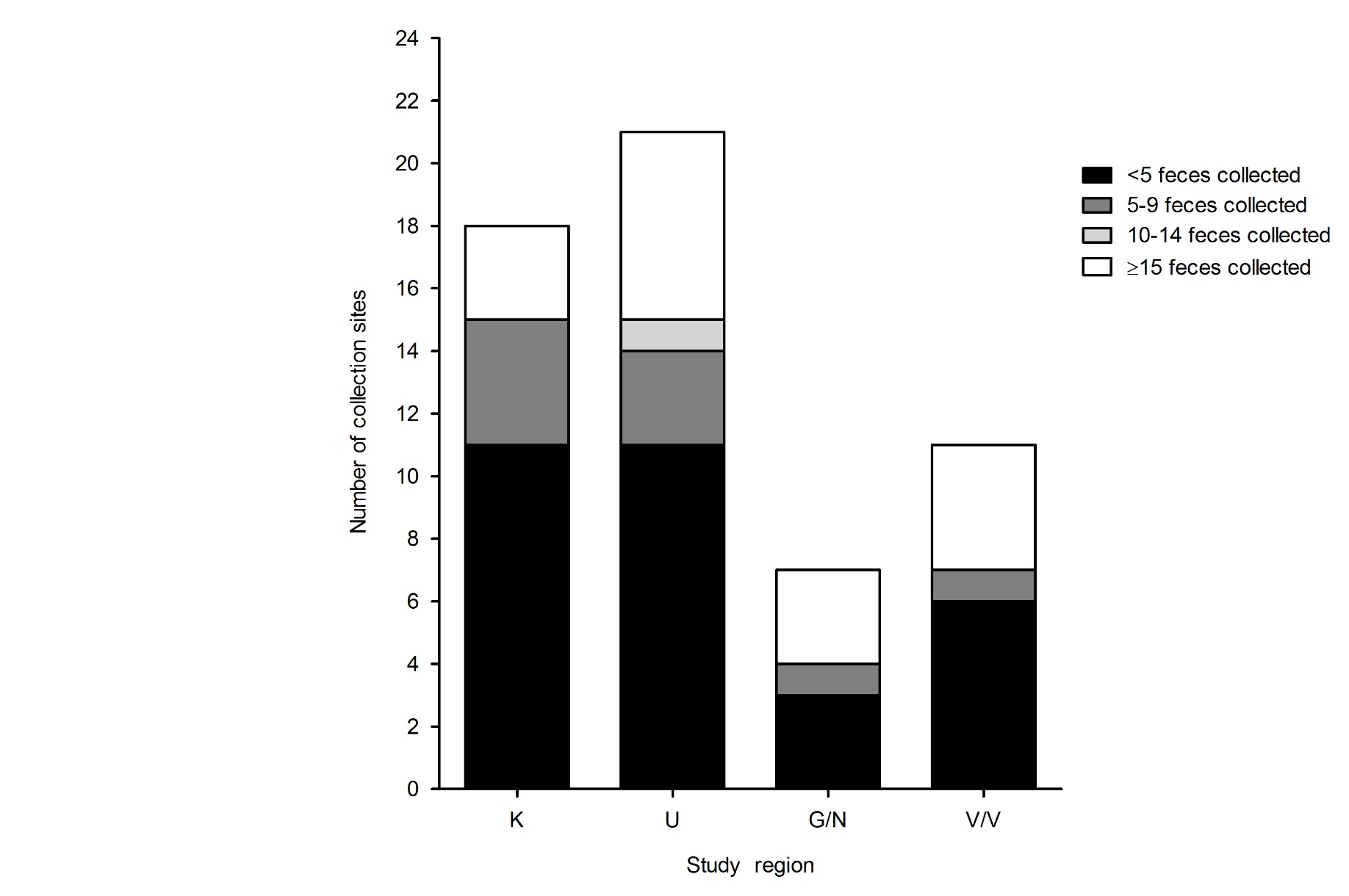


Figure S1. Total number of fox fecal collection sites for each study region 2013-2015. Number of sites where <5 feces (black), 5-9 feces (dark gray), 10-14 feces (light gray), and ≥15 feces (white) collected are shown as part of the total. Study regions are K (Katrineholm), U (Uddevalla), G/N (Gnesta/Nyköping) and V/V (Vetlanda/Växjö).
